# High-density marker profiling confirms ancestral genomes of *Avena* species and identifies D-genome chromosomes of hexaploid oat

**DOI:** 10.1007/s00122-016-2762-7

**Published:** 2016-08-13

**Authors:** Honghai Yan, Wubishet A. Bekele, Charlene P. Wight, Yuanying Peng, Tim Langdon, Robert G. Latta, Yong-Bi Fu, Axel Diederichsen, Catherine J. Howarth, Eric N. Jellen, Brian Boyle, Yuming Wei, Nicholas A. Tinker

**Affiliations:** 1 Ottawa Research and Development Centre, Agriculture and Agri-Food Canada, 960 Carling Ave., Ottawa, ON K1A 0C6 Canada; 2 Triticeae Research Institute, Sichuan Agricultural University, Chengdu, 611130 China; 3 Institute of Biological, Environmental and Rural Sciences, Aberystwyth University, Gogerddan, Aberystwyth, Ceredigion SY23 3EE UK; 4 Department of Biology, Dalhousie University, 1355 Oxford St., Halifax, NS B3H 4R2 Canada; 5 Plant Gene Resources of Canada, Saskatoon Research and Development Centre, Agriculture and Agri-Food Canada, 107 Science Place, Saskatoon, SK S7N 0X2 Canada; 6 Department of Plant and Wildlife Sciences, Brigham Young University, Provo, UT USA; 7 Plateforme d’analyses génomiques, Institut de biologie intégrative et des systèmes, Université Laval, Quebec City, QC G1V 0A6 Canada

## Abstract

***Key message*:**

**Genome analysis of 27 oat species identifies ancestral groups, delineates the D genome, and identifies ancestral origin of 21 mapped chromosomes in hexaploid oat.**

**Abstract:**

We investigated genomic relationships among 27 species of the genus *Avena* using high-density genetic markers revealed by genotyping-by-sequencing (GBS). Two methods of GBS analysis were used: one based on tag-level haplotypes that were previously mapped in cultivated hexaploid oat (*A. sativa*), and one intended to sample and enumerate tag-level haplotypes originating from all species under investigation. Qualitatively, both methods gave similar predictions regarding the clustering of species and shared ancestral genomes. Furthermore, results were consistent with previous phylogenies of the genus obtained with conventional approaches, supporting the robustness of whole genome GBS analysis. Evidence is presented to justify the final and definitive classification of the tetraploids *A. insularis*, *A. maroccana* (=*A. magna*), and *A. murphyi* as containing D-plus-C genomes, and not A-plus-C genomes, as is most often specified in past literature. Through electronic painting of the 21 chromosome representations in the hexaploid oat consensus map, we show how the relative frequency of matches between mapped hexaploid-derived haplotypes and AC (DC)-genome tetraploids vs. A- and C-genome diploids can accurately reveal the genome origin of all hexaploid chromosomes, including the approximate positions of inter-genome translocations. Evidence is provided that supports the continued classification of a diverged B genome in AB tetraploids, and it is confirmed that no extant A-genome diploids, including *A. canariensis*, are similar enough to the D genome of tetraploid and hexaploid oat to warrant consideration as a D-genome diploid.

**Electronic supplementary material:**

The online version of this article (doi:10.1007/s00122-016-2762-7) contains supplementary material, which is available to authorized users.

## Introduction

Crop wild relatives are an important source of genetic variability for improving agricultural species through interspecific crossing and introgression (Zamir 2001). Cultivated oat has been improved through the interspecific transfer of alleles conferring resistance to disease (e.g., Aung et al. 2010; Hoppe and Kummer 1991; Rines et al. 2007), and the transfer of other important quality and agronomic traits to cultivated oat has been initiated or proposed, as reviewed by Loskutov and Rines (2011). However, the use of crop wild relatives for introgression breeding is constrained by interspecific fertility barriers. Many interspecific hybrids are sterile, and those hybrids that can be made often require advanced techniques such as embryo rescue and bridging species, followed by extensive backcrossing, to achieve the transfer of a target allele (Loskutov 2001). Therefore, it is important to develop detailed information regarding the value and transferability of traits from crop wild relatives prior to investing resources into introgression breeding. The genus *Avena* shows a wide variation in genome size, from 4.1 to 12.8 Gbp, which is determined primarily by ploidy level, but may also be influenced by variation in the types and amounts of repeated DNA among genomes (Yan et al. 2016). Differences among genomes of cultivated hexaploid oat and its wild relatives will probably determine the locations into which foreign genes are introgressed, and may also influence the amount of linkage drag or the propensity for chromosomal rearrangements involving introgressed chromosomes. Thus, detailed chromosome-level knowledge of the structural and ancestral relationships among the genomes of *Avena* species is of both practical and theoretical interest.

The genus *Avena* contains up to 30 recognized species (see Table 1, which includes species authorities) in a series of ploidy levels that includes diploids, tetraploids, and hexaploids (Baum 1977; Baum and Fedak 1985a, b; Ladizinsky 1998). A number of the recognized species exhibit high degrees of cross fertility, contrary to the biological species definition (e.g. Ladizinsky 2012). The diploid species fall clearly into two major genome groups: A and C. All hexaploid species, including the cultivated oat, *A. sativa*, are described as having an ACD genome composition, corroborated by fertile interspecific crosses among the hexaploids, as well as by their near-identical genome sizes (Yan et al. 2016). Less clearly, the tetraploids have been assigned designations of CC, AA or AB, and AC or DC. Genomes designated as B and D within the polyploids have not been shown to exist within extant diploid species. Debate remains regarding which A- and C-genome diploids are closest to the A and C genomes of hexaploid oat, and whether or not any extant A-genome diploid is closely related to the B and/or D genomes.

**Table 1:** List of *Avena* species included in this work, classification by genome and PCoA group, and proportion of mapped loci (hexaploid GBS model) present

Species/group^a^	Hap-lome^b^	Ploidy (2*n*)	Mean 2C values (pg DNA)^c^	Num.^d^	Mapped loci (%)^e^	Haplotypes per shared locus^f^
*A. fatua* L.	ACD	6×	25.8	8	91.3	1.58
*Avena* × *glabrata* Hausskn.	ACD	6×	NA	1	81.0	1.17
*Avena* × *haussknechtii* Nevski	ACD	6×	NA	1	73.0	1.02
*A. hybrida* Peterm.	ACD	6×	NA	5	86.1	1.27
*A. occidentalis* Durieu	ACD	6×	25.7	6	90.6	1.45
*A. sativa* L.	ACD	6×	25.7	119	99.0	1.91
*A. sterilis* L.	ACD	6×	25.8	17	94.5	1.69
**Group 1**	**ACD**	**6×**	**25.8**	**157**	**99.7**	**1.96**
*A. insularis* Ladiz.	AC (DC)	4×	18.6	3	58.6	1.14
*A. maroccana* Gand.	AC (DC)	4×	18.5	4	49.0	1.06
*A. murphyi* Ladiz.	AC (DC)	4×	18.7	1	34.2	1.00
**Group 2**	**AC (DC)**	**4×**	**18.6**	**8**	**66.7**	**1.25**
*A. atlantica* Baum et Fedak	A_s_	2×	9.2	3	10.3	1.05
*A. brevis* Roth	A_s_	2×	9.0	6	10.6	1.04
*A. hirtula* Lag.	A_s_	2×	9.1	2	11.1	1.04
*A. hispanica* Ard.	A_s_	2×	8.8	6	10.7	1.04
*A. nuda* L.	A_s_	2×	9.1	6	9.6	1.01
*A. strigosa* Schreb.	A_s_	2×	9.1	16	14.2	1.09
*A. wiestii* Steud.	A_s_	2×	9.1	2	11.2	1.05
**Group 3**	**A**_**s**_	**2×**	**9.1**	**41**	**22.3**	**1.08**
**A** ***. longiglumis*** **Durieu (=Group 4)**	**Al**	**2×**	**9.2**	**3**	**10.4**	**1.05**
*A. canariensis* Baum et al.	A_c_	2×	8.8	5	5.6	1.01
*A. damascena* Rajhathy et Baum	A_d_	2×	8.4	3	6.0	1.01
* A. lusitanica* Baum	A_s_	2×	8.7	2	7.2	1.02
**Group 5**	**A**_**c/d**_	**2×**	**V**	**10**	**V**	**1.05**
*A. abyssinica* Hochst.	AB	4×	16.7	6	12.2	1.04
*A. barbata* Pott ex Link	AB	4×	16.4	5	14.6	1.10
*A. vaviloviana* Mordv.	AB	4×	16.4	4	12.0	1.04
**Group 6**	**AB**	**4×**	**16.5**	**15**	**15.7**	**1.10**
***A. agadiriana*** **Baum et Fedak (=Group 7)**	**V**	**V**	**17.5**	**4**	**10.7**	**1.06**
*A. clauda* Durieu	C_p_	2×	10.3	3	4.2	1.01
*A. eriantha* Durieu	C_p_	2×	10.2	3	5.1	1.02
*A. ventricosa* Balansa ex Coss.	C_v_	2×	10.3	2	4.5	1.00
**Group 8**	**C**	**2**×	**10.3**	**8**	**5.6**	**1.02**
***A. macrostachya*** **Balansa et Durieu (=Group 9)**	**C**_**m**_ **C**_**m**_	**4×**	**21.8**	**1**	**2.1**	**1.02**

^a^Groups and single species that form groups (bold) are based on the established genome subgroup supported by PCoA analysis (Fig. 1). Species that are considered in this work to be homotypic include: *A. nudi*-*brevis* (=*A. nuda*), *A. pilosa* (=*A. eriantha*), *A. ludoviciana* (=*A. sterilis*), *A. byzantina* (=*A. sativa*), *A. prostrata* (=*A. hirtula*) and *A. Magna* (=*A. maroccana*). *Avena* × *glabrata* is a hybrid of *A. sativa* and *A. fatua*, while *Avena* × *haussknechtii* Nevski is hybrid of *A. sativa* and *A. sterilis*, according to Baum (1977). The incorrect use of “*A. nuda”* to refer to a hulless hexaploid species has been corrected to the accepted classification within *A. sativa*. References for authorities are provided by Loskutov and Rines (2011)

^b^Most commonly referenced genome constitution, with alternate speculations in parentheses

^c^Mean genome size reported by Yan et al. (2016). For groups, these values are reported as the unweighted arithmetic mean of species included in the group

^d^Number of accessions after removal of outliers (see Online Resource 1)

^e^Percentage of the total number of loci in the hexaploid GBS model where one or more of the hexaploid haplotypes were found in the respective species or ancestral genome group

^f^Average number of hexaploid haplotypes found at loci where at least one haplotype was found in the respective species or ancestral group

Two points of clarification should be noted regarding the genome designations in oat. First, these designations contain no deliberate implications of orthology to wheat or any other non-*Avena* genomes with the same alphabetical genome designations. Second, there are no established rules on how different two genomes need to be before they are designated by a different letter. So, while the A and C genomes of oat are clearly differentiated, the designations of B and D genomes and the naming of different A-genome sub-types are subject to debate. In this work, we take the viewpoint that these designations can be used for pragmatic purposes if they are informative of a degree of divergence that can be reliably and repeatedly measured.

Structural differences among the A-genome diploid species have been revealed by karyotype analysis (Fominaya et al. 1988; Rajhathy and Thomas 1974) and by the chromosome pairing of their hybrids (Leggett 1989; Thomas 1992), resulting in the division of the A genome into sub-classifications of A_s_, A_c_, A_d_, A_l_, and A_p_ genomes (Table 1). Although no A-genome diploid has shown a perfect karyotype match to the hexaploid A genome, diploids with the A_s_ genome sub-type have shown the highest degree of chromosome pairing in hybrids with hexaploid species (Kihara and Nishiyama 1932; Marshall and Myers 1961; Rajhathy and Morrison 1960). Furthermore, the A_s_ genome species have also shown the closest karyotype matches (Rajhathy and Thomas 1974), in situ hybridization patterns (Chen and Armstrong 1994; Jellen et al. 1994; Linares et al. 1996, 1998) and molecular similarity (Peng et al. 2010; Yan et al. 2014) to the hexaploid species.

Since the D genome of hexaploid oat shows high similarity to the A genome, it has been suggested that the D genome may be a diverged version of the A genome from a recent diploid ancestor that has yet to be identified, or may no longer be extant (Loskutov 2008). Based on evidence from morphological features (Baum et al. 1973), isoenzyme variations (Craig et al. 1974), and fluorescent in situ hybridization (FISH) results (Linares et al. 1998), there is unpublished speculation that the A_c_ genome variant found in *A. canariensis* might be the closest surviving diploid genome to the ancestral D genome donor.

The C genome of hexaploid oat is highly diverged from the A and D genomes, as demonstrated by substantially more pronounced C-banding (Fominaya et al. 1988; Jellen et al. 1993), and these differences have been substantiated by differential FISH analysis (Hayasaki et al. 2000; Jellen et al. 1994; Linares et al. 1998; Yang et al. 1999). There are three C-genome diploid species, which have been further subclassified as having two genome types (C_p_ and C_v_) because of minor structural differences (Rajhathy and Thomas 1967). Based on cytogenetic and molecular evidence, both types have been proposed as being contributors of the C genome in hexaploid species (Chen and Armstrong 1994; Nikoloudakis and Katsiotis 2008; Peng et al. 2010; Rajhathy 1966).

There are four species commonly considered to have AB genomes. Of these, *A. barbata*, *A. vaviloviana*, and *A. abyssinica* may belong to a common biological species, while *A. agadiriana* is distinct (Ladizinsky 2012). Based on FISH analysis using an A-genome satellite probe, Irigoyen et al. (2001) found a clear distinction between A- and B-genome chromosomes within both *A. barbata* and *A. vaviloviana*, justifying their designation as allotetraploids. This was supported by the identification of two different haplotypes of nuclear *Acc*1 genes from AB-genome tetraploids (Yan et al. 2014). In contrast, Chew et al. (2016) found that AB-genome tetraploids clustered closely with A-genome diploids; thus, they suggested that the B genome designation is not justified. Most work, including that of, Chew et al. (2016) has supported a distinction between *A. agadiriana* and other so-called AB-genome tetraploids. *A. agadiriana* differs karyotypically from other AB genome tetraploid species (Badaeva et al. 2010; Jellen and Gill 1996) and molecular analysis has revealed substantial differences between *A. agadiriana* and the *barbata* group (Peng et al. 2010; Yan et al. 2014). This may indicate that *A. agadiriana* contains a different combination of A and/or B genomes from the *A. barbata* group. Since *A. agadiriana* shares similarities with the tetraploids in the AC (DC) genome group (Alicchio et al. 1995), as well as with some hexaploid oats (Badaeva et al. 2010), there is also speculation that this species may contain an AD-genome combination.

It has been proposed that hexaploid oat originated through the formation of an AC genome tetraploid from A and C genome diploids, followed by hybridization to a D genome diploid, with chromosome doubling at each stage to stabilize chromosome pairing (Loskutov 2008; Thomas 1992). This proposal is based partially on the existence of three tetraploid species, *A. insularis*, *A. maroccana*, and *A. murphyi*, which are currently designated as having AC genomes. The AC designation is based on genomic in situ hybridization (Leggett et al. 1994), as well as on the close genetic and morphological proximity of these tetraploids to the hexaploids (Ladizinsky 1971, 1998; Li et al. 2000; Murphy et al. 1968). However, these AC genome designations have been challenged by new molecular evidence (Oliver et al. 2011a, b; Peng et al. 2008; Peng et al. 2010; Yan et al. 2014), which, together with previous observations of meiotic chromosome pairing (Ladizinsky 1969) and FISH analysis (Linares et al. 1998), suggests that these tetraploids may contain the D genome found in hexaploid oat. The validation of the existence of the D genome in tetraploid oat, and a clear molecular delineation between chromosomes of A vs. D origin, would lead to a better understanding and utilization of oat genetic resources.

Many of the above inferences regarding genome evolution in *Avena* have been made using alignments of conserved nuclear or chloroplast genes. Such techniques are favoured because they allow concise estimates of the number and order of genetic mutations within and between genomes (using nuclear genes) or interferences about maternal parents (using chloroplast genes). However, single genes may not represent the average rate of divergence across entire genomes, and may not show adequate diversity to resolve differences within a species or between closely-related species. When reference genomes are available, genome resequencing can be used to construct phylogenies based on many gene sequences. Alternatively, alignments can be built using transcriptomes or captured exomes. However, like many other plants with large duplicated genomes, genome reference sequences are not yet available for species of *Avena*, and large-scale alignments are confounded by the presence of homeologues. As an alternative to these methods, inferences of genetic divergence can be made using composite statistics from molecular marker analysis. Several previous studies of *Avena* using various types of molecular markers have shown a general consensus in the qualitative clustering of species that agrees with most single-gene analyses (Alicchio et al. 1995; Chew et al. 2016; Drossou et al. 2004; Fu and Williams 2008; Li et al. 2000; Nocelli et al. 1999). Of these, only Fu and Williams (2008) used adequate numbers of accessions to address genetic diversity within species, while the most recent study by Chew et al. (2016) is notable for the use of high-density genotyping-by-sequencing (GBS) markers. While molecular markers provide superior genome coverage to single-gene studies, marker assays may confound allelic polymorphism with genetic differences among paralogous loci. Thus, interspecific comparisons using anonymous genetic markers may give biased estimates of distance, particularly when comparing species with different ploidy levels. Furthermore, none of the above studies have used markers with well-characterized map positions that would enable chromosome-based inferences of genetic distance.

Recently, progress has been made in the development of a high-density consensus linkage map in *A. sativa* (Chaffin et al. 2016) based on high throughput array-based SNPs (Tinker et al. 2014) and GBS markers (Huang et al. 2014). This map not only improved substantially upon the previous consensus map (Oliver et al. 2013), but also identified uncertainties and ambiguities in the physical chromosome assignments that had been proposed. As a result, only nine of the 21 hexaploid chromosome representations in the current consensus map are assigned with certainty to physical chromosomes using the most recent chromosome nomenclature proposed by Sanz et al. (2010). However, Chaffin et al. (2016) also illustrated the possibility of assigning chromosomes to genomes based on the similarity of mapped marker sequences to partially sequenced ancestral genomes. We recognized that this concept could be extended through the use of high-density GBS profiling in additional *Avena* species, and that this strategy could help to elucidate a more detailed understanding of the ancestral genomes of hexaploid oat. In preparation for this work, we developed a novel GBS pipeline called ‘Haplotag’ (Tinker et al. 2016) which was designed to discover and assign tag-level haplotypes to discrete diploid loci in a systematic way using population-level filters. We reasoned that, by identifying tag-level haplotypes that contain multiple SNPs, we could achieve a more sensitive detection of ancestral alleles than was possible using single SNP analysis (Tinker et al. 2016).

The objectives of this current work were to examine representative accessions from all available and recognized species of *Avena* using GBS analysis, and to identify their similarities to hexaploid oat based on exact matches to tag-level haplotypes discovered in hexaploid oat. From this, we planned to validate or advance hypotheses about the evolutionary origins of hexaploid oat on a chromosome and map-based level. Furthermore, we sought to explore the allelic diversity of hexaploid oat ancestors on a per-locus basis, leading to recommendations for germplasm conservation and utilization. Because of the above-mentioned limitations of genetic markers, it was not our intention to perform a definitive phylogenetic analysis of the genus *Avena*. Nevertheless, by analysing genetic similarities among a comprehensive set of *Avena* species that is supported by classical phylogenetic studies, we hoped to evaluate the generalized use of GBS in phylogeny analysis and to comment on its potential use in other species where previous phylogenies are lacking.

## Materials and methods

### Plant material

Seeds of 266 accessions representing 27 *Avena* species and two hexaploid oat hybrids were obtained from either Plant Gene Resources of Canada (PGRC) or the United States Department of Agriculture Germplasm Resources Information Network (Table 1 and Online Resource 1). To reduce the potential for misclassification and seed mixtures, at least three accessions per species were assayed when available. Furthermore, some accessions were replicated by sequencing multiple plants from within accessions. The species *A. atherantha*, *A. matritensis*, and *A. trichophylla* (Baum 1977) were not included because of a lack of viable material. Although there remains controversy regarding the distinctness and discovery of *A. maroccana* vs. *A. magna* (Ladizinsky 1994), both species names are used synonymously in the literature for the same accessions, and we have chosen the former. Other species that we considered to be homotypic are noted in Table 1.

### DNA extraction, library construction, and sequencing

Leaf tissue from single plants of each accession was collected in paper envelopes, each containing a 10 g indicating silica gel pouch (silicagelpackets.ca). These were then placed inside large, re-sealable plastic bags until the tissue was dry. Dried leaf tissue was ground in 2 ml plastic tubes containing two 3.1 mm stainless steel beads (Fox Industries, Fairfield, NJ, USA) each using a Tissuelyser bead mill (Qiagen Inc., Mississauga, ON, Canada). DNA was isolated using DNeasy Plant Mini kits (Qiagen Inc., Mississauga, ON, Canada). Complexity reduced, multiplexed GBS libraries were constructed by the Plateforme d’Analyses Génomiques of the Institut de Biologie Intégrative et des Systèmes (IBIS), Université Laval, Quebec City, Canada, based on the *Pst*I–*Msp*I method described by Huang et al. (2014). Complexity reductions were multiplexed using barcode adapters, with 96 samples per pooled library. Sequencing of each pooled library was performed on a single lane of a HiSeq 2500 platform (Illumina, San Diego, CA, USA) using standard Illumina protocols and kits, producing high-output paired-end 150 bp reads. Although 100 bp single-end sequencing was adequate to support the current methods, we chose to perform longer paired-end reads to support future work.

### Generating tag count files and tag-level haplotypes (hexaploid model)

Raw sequence files in FASTQ format were processed using the first step in the UNEAK-GBS pipeline (Lu et al. 2013) to trim the reads, de-convolute the barcodes, and produce a single tag count file for each sample. The tag count files, along with a locus definition file and a haplotype definition file, were used as input for the program Haplotag (Tinker et al. 2016), which was run in ‘production mode’. The latter two files were from previous work where Haplotag was used in ‘cluster discovery mode’ with a cumulative set of 4657 *A. sativa* accessions from multiple studies, intended to develop a consistent set of reference tags from cultivated oat. The full set of 353,133 tags and 164,741 locus assignments from these files are available in Online Resource 2. When Haplotag was run in ‘production mode’ using these input files, the GBS analysis was restricted to the enumeration of tag-level haplotypes that were previously discovered in *A. sativa*. The result was a matrix of samples by loci, with an alphabetical string of tag-level haplotypes assigned to each cell. This was then converted to a binary (plus–minus) matrix, with ‘1’ representing the presence of any tag-level haplotype at a given locus, and ‘0’ representing the absence of any haplotype.

### Sample validation and PCoA of samples (hexaploid model)

To ensure the accuracy of species and genome classifications, the identities of many accessions were validated by estimating genome size using flow cytometry (as described by Yan et al. 2016). Then, all samples were subjected to a principal coordinates analysis (PCoA) of the binary matrix described above based on the Dice dissimilarity index estimated using the package ‘arules’ in the program ‘R’ (R Core Team 2016). Samples with 2C values that conflicted with the accepted ploidy level of the species, and samples that clustered differently from the majority of cohorts within a species, were excluded from further analysis, as noted in Online Resource 1. The PCoA analysis was then repeated to visualize proximity and grouping of samples from validated accessions.

### Merging and normalization of tag counts

Tag count files of validated samples from the same species were merged by summing the counts for the union of all common tags. Following this, the species-level tag count files were down-sampled until all diploid species had the same total tag counts (the sum of tag counts across all tag types = 1×), and tetraploid and hexaploid species had total counts of 2× and 3×, respectively. Down-sampling was performed by generating a new whole-number count for each tag based on a down-sampled expectation. For example, to down-sample a total tag count from 3 × 0^6^ to 2 × 10^6^, a given tag that was observed 600 times would be down-sampled to exactly 400, while a tag that was observed only once would have a 33.3 % probability of becoming zero and a 66.6 % probability of remaining as one. In one special case, the species *A.* *macrostachya* (having only one accession, thus the lowest number of tag counts) was up-sampled using the same methods. Tag count files were also merged and normalized by ancestral groups, as validated by PCoA results, to provide the deepest possible GBS tag count for each ancestral group. These normalized tag count files were used in subsequent analyses, as noted.

### Genotype analysis from de novo haplotype discovery

A second GBS analysis was performed based on *de novo* haplotype discovery using the normalized species-level tag counts. First, all tags that were observed between 20 and 50 times within a species were identified as ‘key tags’. The lower limit (20) was chosen to favour tags that were represented efficiently in the sequenced GBS library, while the upper limit was set to avoid including tags from repetitive elements. To reduce ascertainment bias further, the number of key tags contributed by each species was truncated to that of the species with the smallest number of key tags. Following this, the remaining key tags were joined by union to form a set of key tags intended to represent all species. Next, a plus–minus matrix of key tags by species was tabulated from the normalized tag count files, this time counting any number of observed tags (>0) within a species as present. These data were subjected to PCoA analysis using the Dice dissimilarity index, as described earlier.

### In silico polyploid analysis

To infer potential polyploidization events from extant species, in silico hexaploids were created by merging normalized tag counts from selected diploid and tetraploid species. Since ploidy was already compensated for in the normalized tag count files (a diploid contributed 1× tags and a tetraploid contributed 2×), the total number of tags in the derived hexaploids were all equal (at 3×) and required no further normalization. All possible combinations of one A-genome diploid species plus one AC (DC) genome tetraploid species, as well as one C-genome diploid species plus one AB genome tetraploid species, were generated. Genetic similarities among extant and in silico hexaploid species were estimated using the Dice dissimilarity index based on the plus–minus scores from the de novo GBS model, and a PCoA analysis was conducted as described above. Hierarchical clustering by the unweighted pair group method with arithmetic mean (UPMGA) was also used to explore the proximity of extant and in silico species. A similar strategy was employed to explore in silico tetraploids formed from extant diploids.

### Comparative chromosome E-painting by species and ancestral group

In previous work, markers discovered in the Haplotag hexaploid model were placed on the hexaploid consensus map using the method described by Chaffin et al. (2016). This produced an expanded linkage map called ‘Expanded Oat Consensus Map 2016’ containing 50,668 loci, which is available for download from the public oat database ‘T3/Oat’ (https://triticeaetoolbox.org/oat/). From this, a mapped subset of the loci and tag-level haplotypes from the complete hexaploid model was selected (Online Resource 2). These tags, along with the normalized tag counts from each species and each ancestral group (as defined by PCoA), were used in a Haplotag production model to generate a text-based matrix (genotypes represented by character strings) and a plus–minus matrix (presence-absence of hexaploid haplotypes recorded as ‘1’ or ‘0’). The overall density of shared hexaploid tag-level haplotypes for each species and ancestral group was calculated as the frequency of ‘plus’ values in the plus–minus scoring matrix. A haplotype diversity index was also computed by counting all characters in the text-based scoring matrix and dividing by the number of ‘plus’ values in the plus–minus matrix. The scoring matrices were then smoothed and scaled using a sliding 30 cM window to summarize the chromosome region surrounding each 1 cM increment of the consensus map. The frequency of loci with ‘plus’ scores (locus-presence) was recorded within each window, and the average number of haplotypes per ‘plus’ locus (haplotype-diversity) was calculated.

The smoothed locus-presence and haplotype-diversity profiles were then interpreted using electronic painting (E-painting) on circle graphs of the oat consensus map to show where each species or ancestral group exceeded a given threshold. These thresholds were intended to delineate the relative frequency within a given species or group rather than the absolute frequencies. Since an idealized diploid would contain 1/3 of the hexaploid haplotypes and an AC (DC) tetraploid would contain 2/3, we set these thresholds to show where locus presence or haplotype diversity exceeded the 33rd or 66th percentiles within each species or group, respectively. For the haplotype-diversity profiles, these thresholds were adjusted slightly to achieve better differentiation among chromosomes. Circle diagrams were created using ‘Circos’ software Version 6.7 (Krzywinski et al. 2009).

## Results

### Raw data and GBS tags

Raw data and tag sequences from the GBS analyses are recorded in Online Resource 2, as described below and in the first index sheet of the online resource. All of the 353,133 tags assigned to 164,741 hexaploid loci (Sheet 1) were used to generate a presence-absence matrix of loci-by samples (Sheet 2). Of these, 26,910 loci placed on the hexaploid oat consensus map (Sheet 3) were used in the map-based chromosome E-painting. In the *de novo* analysis, a total of 85,831 unique tag-level haplotypes (Sheet 4) were scored across species and in silico polyploids (Sheet 5).

### Validation of accessions

Initially, a set of 672 samples representing 266 accessions was examined using PCoA analysis. Based on this result, as well as on flow cytometry and phenotypic observations (not shown), 40 samples were considered to be misclassified and were removed, leaving 632 samples from 247 accessions for further analysis. A list of the samples that were removed is shown in Online Resource 1, since this information may assist with future genebank curations. For each of these accessions, we provide a possible reclassification by species and/or genome group; however, caution should be applied in using this information. In some cases, where we tested multiple seeds from an accession, we observed heterogeneous results. So, although these misclassifications represent a small proportion of accessions compared with those considered to be correct, the heterogeneity of species classifications that we observed in some accessions could potentially exist within a few correctly classified accessions where we tested only one sample. A deeper analysis of within-accession heterogeneity will be the subject of a forthcoming study.

### Genomic relationships among *Avena* species

**Fig. 1:**
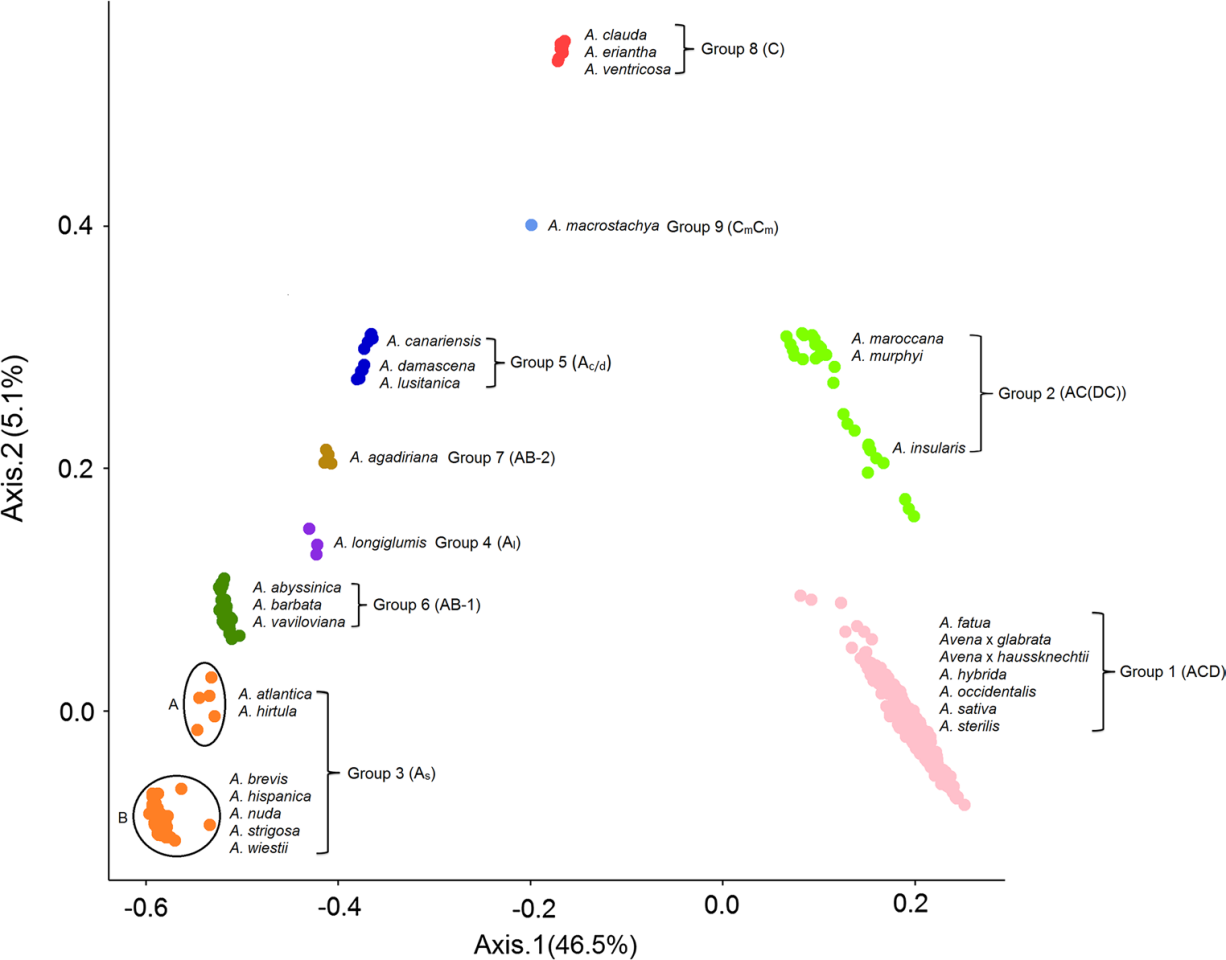
Hexaploid-based PCoA analysis depicting the relative distances between hexaploids and other *Avena* accessions used in this study on the first two axes. Each *point* represents a sample derived from a single seed from an accession. The number of accessions for each species is shown in Table 1. Some accessions are represented by multiple samples (see Online Resource 1) (colour figure online)

Relationships among *Avena* species were visualized using PCoA analysis on individual samples based on mapped loci (Fig. 1) as well as on a per species basis using *de novo* haplotypes (Online Resource 3). Both analyses revealed similar groupings by species, supporting a consistent nomenclature of nine different ancestral groups (Fig. 1; Table 1; and Online Resource 3). Because the map-based GBS analysis was conducted on a per-sample level, and because it provided the clearest separation among species and ancestral groups, we will describe those results, drawing attention to the *de novo* analysis only where results were less consistent.

All of the hexaploids formed a homogeneous group (Group 1) that was separated from other species. Three tetraploid species, *A. insularis*, *A. maroccana*, and *A. murphyi* clustered together into Group 2, and showed the highest similarity (66.7 %) to hexaploid oats based on shared haplotypes (Table 1). Within this group, *A. insularis* showed a higher frequency (58.6 %) of shared hexaploid haplotypes than *A. maroccana* (49.0 %) or *A. murphyi* (34.2), and appeared to cluster most closely with the hexaploid accessions in both analyses (Fig. 1 and Online Resource 3), suggesting that *A. insularis* is the non-hexaploid species most closely related to hexaploid oats.

The A-genome diploid species showed the highest overall diversity and formed several well-separated groups. All of the A_s_-genome diploid species, with the exception of *A. lusitanica*, formed a relatively broad cluster (Group 3) which could be further divided into two sub-groups in the map-based analysis (Fig. 1): Group 3A contained two A_s_-genome species (*A. atlantica* and *A. hirtula*), with the rest forming Group 3B. In the *de novo* analysis (Online Resource 3), the species *A. atlantica* and *A. hirtula* were also slightly separated from the remainder of Group 3. Of the remaining A-genome species, the A_l_-genome species *A. longiglumis* formed a well-separated group (Group 4), whereas the A_c_-genome species *A. canariensis* and the A_d_-genome species *A. damascena* formed a single cluster (Group 5), along with two accessions from *A. lusitanica*.

The AB-genome tetraploids formed two separate clusters (groups 6 and 7) that were distinct from the A-genome groups, but within the same region. Group 6 was comprised of three species (*A. abyssinica*, *A. barbata,* and *A. vaviloviana*), while Group 7 contained only one species, *A. agadiriana* (Fig. 1). The separation of AB from A genome groups was not as obvious in the de novo analysis, but the two ploidy levels were still separated effectively by the second axis (Online Resource 3).

All three diploid C-genome species fell into a single well-separated cluster (Group 8). This group showed the greatest distance from the A_s_ genome (Group 3), based on the first two PCoA axes. The perennial tetraploid species *A. macrostachya* was separated from all other species (Group 9), but showed the closest relationship with the C-genome diploids.

### Analysis of in silico polyploids

**Fig. 2:**
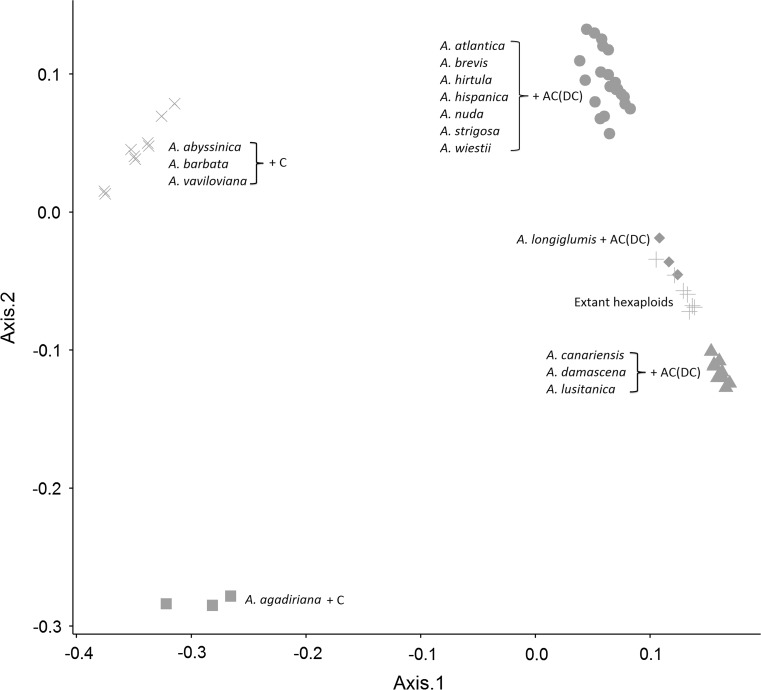
PCoA analysis including all extant hexaploids and theoretical in silico hexaploid species generated by combining tag counts from diploid-plus-tetraploid combinations. The *symbols* and *colours* represent in silico combinations that clustered together, as labelled. The *blue* “+” symbols represent all extant hexaploid species analysed in this study (colour figure online)

Fifty-one possible combinations of diploids with tetraploids were used to create in silico hexaploids, and pair-wise genetic similarities were estimated among all extant and in silico hexaploids. The consistent PCoA patterns shown by multiple species within most ancestral groups provides an effective replication of these results at the ancestral-group level (Fig. 2). All in silico hexaploids comprised of AC (DC) genome tetraploids plus A-genome diploids clustered closely with the extant hexaploids. The closest in silico matches to the extant hexaploids included the AC (DC) tetraploids plus *A. longiglumis*, suggesting a possible role of these species in the formation of the hexaploids. The in silico hexaploids comprised of AB genome tetraploids and C-genome diploids formed a cluster that was more separated from the extant hexaploids. Hierarchical cluster analysis (Online Resource 4) suggested that the extant hexaploids are most closely related to predicted in silico hexaploids comprised of the AC (DC) genome tetraploids and the A_s_ or A_l_ genome diploids.

**Fig. 3:**
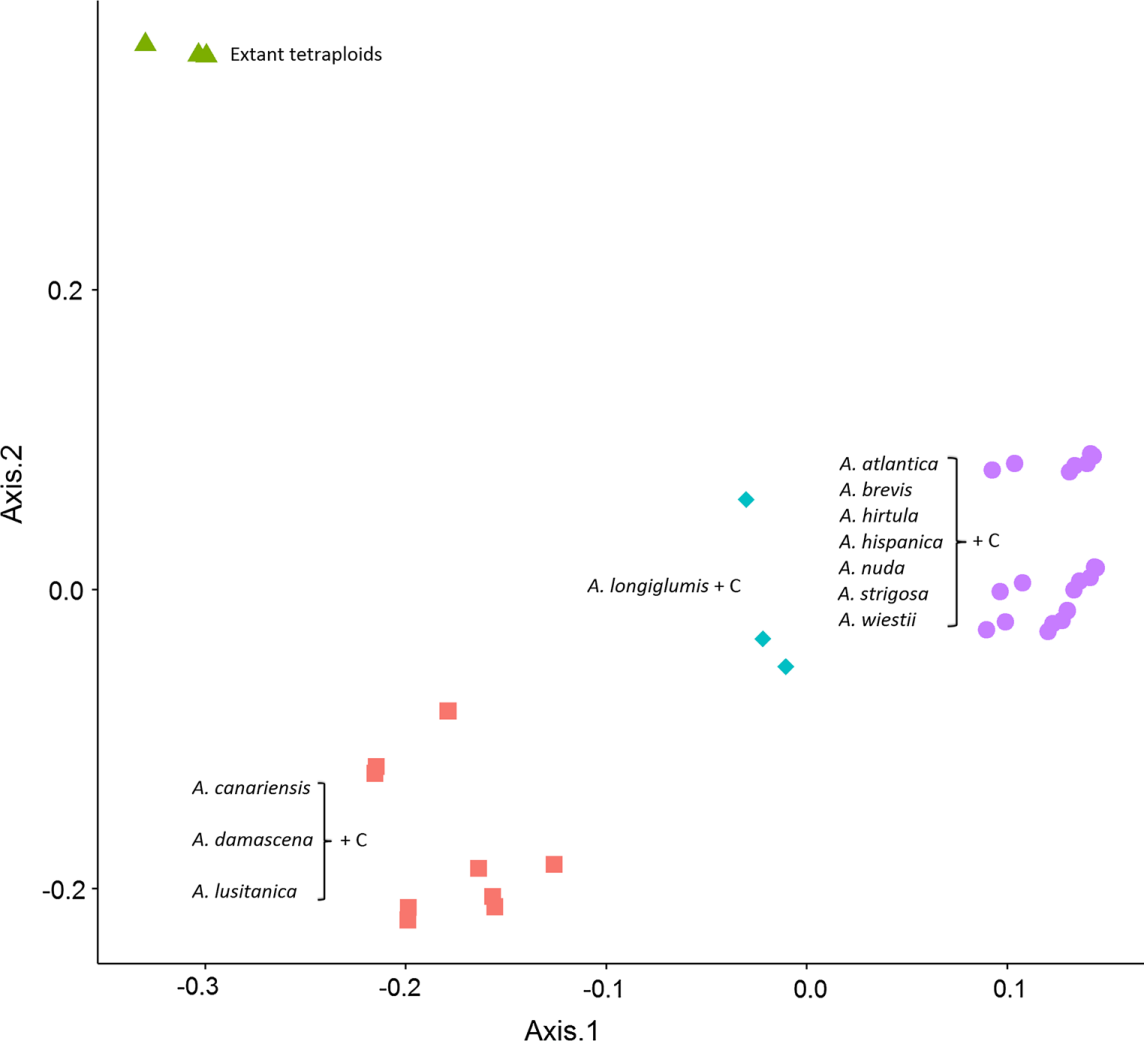
PCoA analysis including all extant AC (DC) tetraploids and theoretical in silico tetraploid species generated by combining tag counts from A-genome diploid plus C-genome diploid combinations. The *symbols* and *colours* represent in silico combinations that clustered together, as labelled. The *green triangles* represent all extant AC (DC) tetraploid species analysed in this study (colour figure online)

Thirty-six in silico tetraploids combining all extant A- and C-genome diploids from this study were created to identify putative ancestral diploids involved in the formation of the AC (DC) genome tetraploids. PCoA analysis (Fig. 3) and hierarchical clustering (Online Resource 5) showed that none of the predicted in silico tetraploids clustered together with the extant AC (DC) tetraploids, suggesting that diverged, non-extant forms of the A and/or C genomes were involved in the formation of these tetraploids.

### Chromosome E-painting by density of shared hexaploid haplotypes

**Fig. 4:**
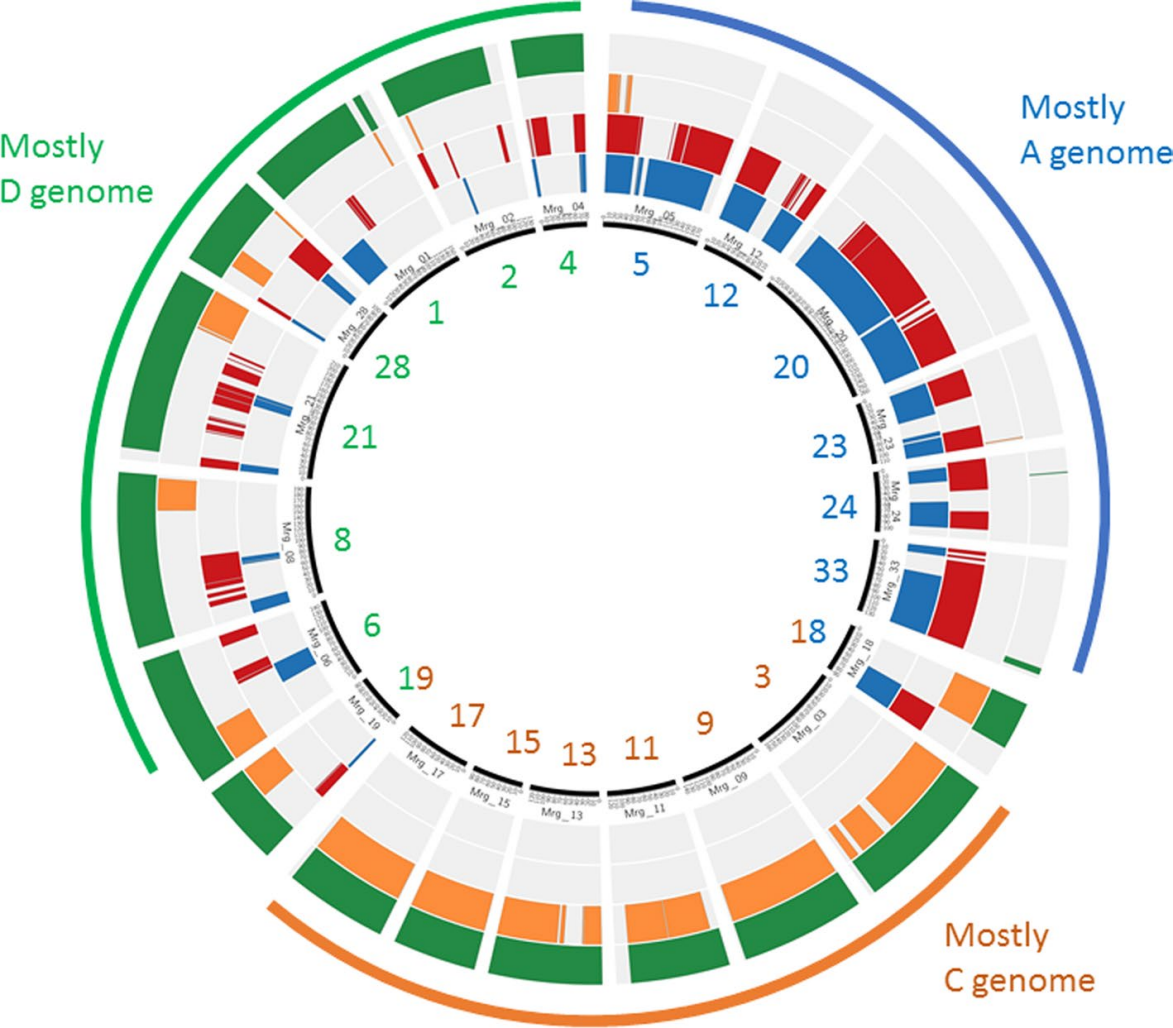
E-painting of chromosome representations in the hexaploid oat consensus map. The 21 chromosome representations with Mrg identification numbers described by Chaffin et al. (2016) are shown as elements of the circle, scaled by cM distance, and ordered by inferred genome origin. *Four concentric rings of different colours* indicate which chromosomes within the map contain a relatively high frequency of matches to the ancestral group represented by the respective ring: *blue* (*innermost ring*) represents A-genome diploids; *red* (*second ring*) represents the A_c/d_-variant genome of the group containing *A. canariensis*, *A. damascena* and *A. lusitanica*; *orange* (*third ring*) represents C-genome diploids; and *green* (*outermost ring*) represents AC (DC)-genome tetraploids. The three outermost arcs are an interpretation of the genome contributions to the majority of chromosomes within each arc, based on hybridization pattern to the ancestral genomes (colour figure online)

Unscaled heat maps, based on the sequential map position of each tested locus, show details of shared hexaploid haplotypes within each species and genome group (Online Resource 6, Sheet 1). These heat maps were smoothed and scaled using a sliding 30 cM window (Online Resource 6, Sheet 2). Thresholds were set to allow chromosome E-painting based on relative hexaploid similarity within all ancestral groups (Online Resource 7) and four groups were selected as having the greatest differential resolving power among hexaploid chromosome representations (Fig. 4). We emphasize here that the thresholds are set independently for each ring in the above figures, since the overall similarity of some groups is much higher than others (Table 1) and the intention here is to observe patterns of relative similarity within each group.

In Fig. 4 and Table 2, we have interpreted the painted chromosome representations by ordering them as follows: chromosomes with Mrg identifiers 5, 12, 20, 23, 24, 33, and part of 18 showed the strongest relative matches in the A_s_-genome diploid species. These same chromosomes showed weak relative matches in the C-genome species and also in the AC (DC) group. Table 2 illustrates that this group of chromosomes is consistent with those that have been confirmed by monosomic assignment as being A-genome chromosomes. Chromosomes with Mrg numbers 3, 9, 11, 13, 15, 17, and parts of 18, 19, 6, 8, 21, and 28 showed the weakest matches to A_s_-genome diploids, and the strongest matches to C-genome diploids and the AC (DC) group. Table 2 illustrates that this group of chromosomes is consistent with those that have been confirmed by monosomic assignment as being C-genome chromosomes. Chromosomes with Mrg numbers 1, 2, and 4, as well as parts of 19, 6, 8, 21, and 28 showed relatively weak similarities to A and C genome diploids, but strong similarities to the AC (DC) tetraploids. Table 2 illustrates that this group of chromosomes is consistent with those that have been confirmed by monosomic assignment as being D-genome chromosomes. In Online Resource 7, it can be seen that all other A-genome groups, as well as the AB genome groups, follow a similar pattern to that of A_s_.

**Table 2:** Previous and current physical assignment of 21 chromosome representations from the hexaploid oat consensus map

Consensus chromosome^a^	Confirmed assignment^a^	Previous assignment^b^	Diploid assignment^c^	New genome assignment^d^
Mrg05	16A	**16A**, 1C	AD	A
Mrg12		13A	AD	A
Mrg20		19A	AD	A
Mrg23	11A	**11A**	AD	A
Mrg24		8A (14D)	AD	A
Mrg33		15A	AD	A
Mrg18		7C–17A	C	C/A
Mrg03	4C	**4C** (10D)	C	C
Mrg09	6C	**6C**	C	C
Mrg11		1C	C/A	C
Mrg13		(20D)	C	C
Mrg15	2C	**2C** (10D)	C	C
Mrg17		3C	C	C
Mrg19	21D	**21D**	C/AD	C/D
Mrg06		14D	AD/C	D/C
Mrg08	12D	**12D**	AD/C	D/C
Mrg21		(16A)	AD/C	D/C
Mrg28		7C–(17A)	C	D/C
Mrg01		(5C)	AD	D
Mrg02	9D	**9D**	AD	D
Mrg04	18D	18D	AD	D

^a^Consensus chromosome representation and confirmed assignments are based on Chaffin et al. (2016)

^b^Chromosome assignments by Oliver et al. (2013) were based on an earlier consensus map with fewer markers. Bold font assignments are now confirmed, those in parentheses are probably incorrect, and those remaining were not confirmed but are not disputed

^c^Diploid genome assignment was performed by Chaffin et al. (2016) based on sequence matches to draft shotgun genome sequences of A- and C-genome diploid oats. This method was unable to resolve A and D genome chromosomes. Where assignments are split by a forward slash, the assignment given to the longest part of the chromosome is shown first

^d^Inferred genome assignment based on the current study. Where assignments are split, suggesting a large inter-genome translocation, the assignment given to the longest part of the chromosome is shown first. Possible inter-genome translocations smaller than 15 % of a chromosome are not shown in this table but can be inferred based on Fig. 1

### Chromosome E-painting by haplotype diversity

The highest diversity of hexaploid haplotypes was observed in the AC (DC) group, with an average of 1.24 haplotypes for those loci where any hexaploid haplotype was present (Table 1). This was much higher than the diversity seen in any other group, with the exception of the ACD genome group in which the haplotypes were originally discovered. Of the AC (DC)-genome tetraploids, the species *A. insularis* shared more hexaploid alleles (1.14 alleles per locus present) than either *A. maroccana* (1.06) or *A. murphyi* (1.00) (Table 1). These data were also interpreted using a circle diagram, with independent thresholds for each ancestral group to show relative patterns across chromosomes (Online Resource 8). Although not as striking as the patterns seen for locus presence (Fig. 4), the diversity of hexaploid haplotypes followed a similar pattern that matched the genome assignments made earlier.

## Discussion

This work represents one of the largest and most comprehensive genomic studies of the genus *Avena* to date, and has generated the following novel results, which are discussed below: (1) support for the definitive classification of DC-genome tetraploids, (2) a map-level identification of the ancestral origin of 21 hexaploid chromosomes, (3) confirmation that no extant A-genome diploids should be considered as D-genome diploids, and (4) evidence for the continued classification of a diverged B genome in AB tetraploids. We begin with a discussion to support the chosen methods of data analysis.

### Justification and comparison of two methods of GBS analysis

Although molecular markers have the capacity to estimate both within- and among-species diversity, with genome coverage that is superior to single-gene methods, it is recognized that GBS and other marker technologies present limitations and potential sources of bias relative to alignment-based phylogeny analyses (DaCosta and Sorenson 2016; Qi et al. 2015). In particular, the use of GBS in the absence of a reference genome may confound intra- vs. inter-locus polymorphism, and the filtering of polymorphisms based on allele or heterozygote frequency could also introduce bias. Since we are not attempting to estimate magnitudes of divergence, we have accepted a pragmatic tolerance of these factors. Furthermore, the methods that we have selected are deliberately focused on distinguishing among species that contain a series of homeologous genomes, such that variation among paralogous loci must be incorporated. We also attempted to address potential sources of bias using two different methods of GBS analysis; thus, providing two independent assessments of marker-based diversity. In one analysis, we calculated genetic similarity based on the presence of tag-level haplotypes that were mapped in the hexaploid genome. In the second analysis, we attempted to avoid potential ascertainment bias by estimating similarities based on the sharing of key GBS tag-level haplotypes. In this analysis, we mitigated the problem of allele dropouts by combining and normalizing tag counts by species, to achieve a high effective sampling depth. The fact that both methods produced clear and qualitatively consistent separation among species should lend credibility to those parts of our discussion that relate to phylogeny.

It is interesting that the map-based hexaploid analysis produced greater separation among many species than did the *de novo* discovery method. We had expected to find better separation using the latter method, which should be more inclined to identify differences based on unique haplotypes found only in one species. One potential explanation for our result is that some *A. sativa* oat varieties contain introgressions from other species (e.g., Aung et al. 2010; Hoppe and Kummer 1991; Rines et al. 2007); thus, it is likely that some mapped hexaploid haplotypes have recent origins in other species. However, this would probably account for only a small number of introgressions from *A. sterilis*, *A. strigosa*, and *A. maroccana*, and could not account (for example) for the improved separation seen between A- vs. AB-genome species in the hexaploid GBS model. A more likely explanation is that the strong separation among species in the hexaploid analysis is caused primarily by differing degrees of overall similarity to the hexaploid genome. This would also explain why the clusters in the hexaploid model (Fig. 1) are positioned along a continuous arc, reflecting a gradient of similarity on two axes, whereas the clusters in the *de novo* model (Online Resource 3) follow a less linear pattern. It is likely that the two axes in Fig. 1 are correlated with markers from the A or D genomes (axis 1) and the C genome (axis 2).

### The ancestral D genome of hexaploid oat can be found in three tetraploid species

We consider the most important result from this work to be three strong lines of evidence that the C and D genome of hexaploid oat originated from an ancestor that is similar to the three extant DC genome (formerly AC) tetraploid species. First, our results from the analysis of in silico polyploids showed that complementation of the AC (DC) genomes with any A-genome diploid resulted in close theoretical matches to the extant ACD hexaploids. Moreover, these matches were much better than any match involving two A genomes plus a C genome. If the tetraploids contained an AC genome, the match of hexaploids to AC + A would not have been substantially better than the matches to A + A + C.

The second line of evidence is provided by map-based chromosome E-painting. This approach was effective likely because most GBS tags originate in non-coding DNA (Huang et al. 2014) that is subject to rapid divergence among genomes that have become separated by reproductive barriers. Thus, the A and D genomes could be distinguished by a composite analysis of GBS tags more effectively than by single conserved gene sequences. In the analysis depicted by Fig. 4, approximately 1/3 of the hexaploid chromosomes matched relatively poorly to A- and C-genome diploids, but showed strong matches to the three AC (DC) tetraploid species. These were designated as D-genome chromosomes. Conversely, the 1/3 of chromosomes not matched by the AC (DC) tetraploids showed strong matches to the A-genome diploids, and were designated as A-genome chromosomes. These assignments were consistent with all previously confirmed physical assignments made by Chaffin et al. (2016) confirming that the AC (DC) tetraploids contain a D, and not an A, genome.

A third line of evidence is that the diversity of hexaploid alleles in AC (DC) tetraploids was higher than for any other species, especially for the chromosomes determined to be of C- and D-genome origin in the above analysis (Online Resource 8). This also suggests that the ACD and DC species share a recent common DC ancestor, and possibly, that multiple polyploidization events occurred to maintain the multiple shared haplotypes that were observed at many loci.

All of the above results support a final re-designation of *A. insularis,* *A. maroccana,* and *A. murphyi* as DC genomes. This would reflect a true A vs. D differentiation in hexaploid genomes, as well as a recognition of the probable common lineage of DC and ACD species to an ancestral D genome that is substantially diverged from extant A genomes. It is recognized (and discussed later) that the D genome is related to the A genome, and that it may have diverged from A-genome ancestors within the polyploid species. However, this proposition, together with our results showing a clear similarity of D-genome chromosomes between the DC and ACD genome species, provides evidence for a sequence of polyploidization events where the A genome is the most recent hexaploid addition to a common DC-genome ancestor.

This is not the first suggested use of the DC genome designation, nor the first evidence that has been presented for this nomenclature (Yan et al. 2014). However, most previous reports, including the recent report by Chew et al. (2016), have continued to identify the tetraploids as AC genomes. This classification fails to identify the closest ancestry of the D genome chromosomes that are recognized in hexaploid oat. Our new evidence and call for the DC genome designation is relevant and important for ongoing germplasm conservation efforts, as well as for future efforts in pre-breeding and cultivar development. It means that the D genome is available in extant non-hexaploid wild relatives for comparative genome analysis and allele mining. Many QTL in cultivated oat have been reported on chromosomes that correspond with those from the D genome, including the N1 locus responsible for primary variation in hull retention (De Koeyer et al. 2004), now known to be on Mrg21, and a major flowering time QTL on Mrg02 (Klos et al. 2016). Preservation, expansion, and utilization of DC genome tetraploids, especially *A. insularis,* which showed the highest number and diversity of hexaploid haplotypes, should be considered a high priority.

### Physical chromosome assignment in the hexaploid consensus map is confirmed and improved

Our chromosome E-painting results have also contributed to a substantial improvement in physical chromosome assignment in the hexaploid linkage map (Table 2). Here, we use the most recent nomenclature for physical chromosomes (Sanz et al. 2010), which merges two previous physical nomenclatures, compared to the consensus map nomenclature of Chaffin et al. (2016). We have shown that all of the nine validated chromosome assignments are consistent with the current analysis; thus, we have used the current analysis to assign genome designations to all of the remaining chromosomes. This includes the designation of two important homeologous chromosomes that were previously ambiguous: Mrg20 is from the A genome and Mrg21 is mostly from the D genome.

Because these assignments were made within chromosomes on a map-based level (Fig. 4), we can also evaluate some of the genome restructuring that has taken place within the hexaploid genome. The majority of translocations appear to involve the D and C genomes, possibly as non-reciprocal translocations, since there appear to be six intact C chromosomes vs. three D chromosomes that do not contain significant translocations from the C genome. This observation is consistent with FISH results reported by Irigoyen et al. (2002) and Sanz et al. (2010), who both observed a majority of C- to D-genome translocations. The chromosome representations Mrg18 and Mrg28 were previously assigned as the reciprocal translocations 7C–17A/17A–7C (Oliver et al. 2013), although this designation was not confirmed by Chaffin et al. (2016). Of these, only Mrg18 contains a clear A/C translocation, while Mrg28 appears to contain a C/D translocation. While it is possible that non-balanced A/C/D translocations might have occurred, this substantial change in interpretation will require further evaluation. The preponderance of C/D vs. C/A or A/D translocations may provide further evidence that the C and D genomes joined first to form a DC tetraploid, and that the A genome of the hexaploid was introduced much later, possibly after the C and D genomes in the tetraploid had co-evolved, and after the A and D genomes had diverged substantially from a common ancestor. However, additional factors may also have affected the differential fragmentation of the three hexaploid genomes. One such factor may be the tolerance of each genome to fragmentation, which may relate to systematic changes in expression and suppression that took place following polyploidization events. Such factors require further investigation.

While this map-level analysis of translocations may provide valuable information for future genome analysis and sequence assembly, it should also be emphasized that: (1) this consensus map does not (by definition) represent the genome configuration of all oat varieties (Chaffin et al. 2016), (2) errors and uncertainties are present in all linkage maps, and (3) the presence of translocations is known to be heterogeneous among oat varieties (Irigoyen et al. 2002; Jellen and Beard 2000; Sanz et al. 2010).

### Diploid species containing the A genome are diverse, but none contain the hexaploid D genome

At least twelve diploid species containing the A genome have been proposed to be part of the genus *Avena*, of which we have studied eleven. Our results showed this to be the most diverse set of species within a single genome designation (Fig. 1 and Online Resource 3), which agrees with the broad geographic distribution of the A-genome diploids (Baum 1977). Of interest is the result from in silico polyploid analysis suggesting that *A. longiglumis* could be the A genome donor, combined with a CD genome from one of the tetraploids (Fig. 2). Separate groups within the A-genome designation also matched well with the presence of previously reported structural differences among these genome types. The species in Group 5, including *A. canariensis* as well as some *A. damascena* and *A. lusitanica* accessions, showed the greatest divergence from other groups (Fig. 1 and Online Resource 3). The two species *A. canariensis* and *A. damascena* form hybrids with regular bivalent pairing in most pollen mother cells (Leggett 1984) and, although *A. lusitanica* is often classified as having an A_s_ genome, this was questioned by Thomas (1992) and its clustering here, rather than with other A_s_ species, may support a future reclassification. Both *A. canariensis* and *A. damascena* have been proposed to be the D-genome donors of the hexaploid species, not only because of the absence of an A-genome specific DNA repeat (Linares et al. 1998), but also because of considerable divergence from other A-genome diploid species (Baum et al. 1973; Rajhathy and Baum 1972). While this divergence is supported strongly by our results, our map-based analysis of hexaploid haplotype identity suggests that the A_c_ and A_d_ genomes are much closer to the hexaploid A genomes than they are to the hexaploid D genomes (Fig. 4). Our in silico tetraploid analysis (Fig. 3) supports this conclusion.

### Diploid species containing the C genome are genetically homogeneous

There are three diploid species considered to carry the C genome. All three of these were included in the Section *Ventricosa* (Baum 1977). On the basis of karyotype, they were further divided into two genome types: C_p_ (*A. clauda* and *A. eriantha*) and C_v_ (*A. ventricosa*) (Leggett and Thomas 1995; Rajhathy and Thomas 1967). In this study, all three C-genome diploid species were tightly clustered based on two PCoA axes (Fig. 1 and Online Resource 3), suggesting a high degree of genetic homogeneity. This was consistent with other molecular studies (Drossou et al. 2004; Yan et al. 2014), with the fertility of interspecific hybrids (Rajhathy and Thomas 1967), and with additional karyotype studies (Fominaya et al. 1988; Rajhathy and Thomas 1967). The geographical distribution of this group may also explain the high degree of genetic homogeneity among these species. All species within this group are restricted to the Mediterranean shoreline (Baum 1977). Both C_p_-genome species are distributed in nearly identical regions around the Mediterranean, while the range of the C_v_-genome diploid *A. ventricosa* is within that of the C_p_-genome species (Rajhathy and Thomas 1967). Other than through morphological differences (Baum 1977), it would be difficult to support the current classification of three C-genome species.

### The B genome has diverged from the A genome and there are two distinct AB-genome groups

Our results are in agreement with most other literature in suggesting that the B genome within the AB-genome tetraploids is sufficiently different from the A genome to cause a distinct clustering of AB-genome species. However, Chew et al. (2016) observed that each of two different AB-genome groups coincided with an A-genome group. We have since determined that the majority of the A-genome members of their Group 4A (including CN 25414, PI 657427, PI 657472) are probably misclassified tetraploids (see Online Resource 1); thus, it is possible that this group is exclusively a tetraploid group. Such misclassifications are usually initiated by the people who originally submitted the accessions to a genebank, and are not the fault of the genebank curator or the users through whom these errors propagate. Nevertheless, while we consider that an A vs. B distinction may still be warranted, we agree that the B genome is likely to be a diverged form of the A genome, and that close relatives of both the A and B genomes may be found within the A genome group. Such work may require further genotyping, since our efforts to identify components of the AB genome through in silico methods were inconclusive (data not shown).

In this study, *A. agadiriana* was separated from the other AB genome tetraploids (Fig. 1), which confirms previously reported structural differences. However, *A. agadiriana* was substantially more similar to the AB group than it was to the AC (DC) group. Furthermore, based on E-painting (Online Resource 7), *A. agadiriana* appears similar only to the A genome of hexaploid oat and not to the D genome, in the same manner as the other AB genome tetraploids. Because of the proximity of *A. agadiriana* to the group containing *A. canariensis*, we tentatively propose that it may share one of its two genomes with this group. The existence of two distinct AB-genome groups, and their similarity to A-genome diploid groups, suggests that further detailed work should be performed to gain insight into the formation of these tetraploids.

### Further research possibilities

Through this work, we have demonstrated that GBS analysis is effective in the qualitative delineation of genome similarities at multiple levels, including ancestral groups, species, and accessions within species. Although this work has focussed primarily on distinctions at the species level, we noted in the development of these methods that these data could be reanalysed to focus on greater resolution within each species, and we intend to pursue this in forthcoming work. In addition, we have demonstrated a method by which genome origins can be effectively delineated at a map-based level in hexaploid oat. This method may be useful in other complex polyploids for which genome sequences are not yet available. We found that GBS analysis was robust, with two different approaches giving similar results at this level of exploration. However, we caution users regarding potential pitfalls in GBS analysis, including ascertainment bias caused by filtering and estimation bias in GBS allele frequencies for phylogenetic analysis. While GBS was effective for the purposes of this work, additional research is required to calibrate estimates of similarity based on GBS with evolutionary distance. Such work will probably require the assignment of GBS tags to draft genome sequences, permitting the alignment and analysis of long and accurate GBS haplotypes. We also suggest that GBS analysis may contribute sequence data that could be used to design new genome- or chromosome-specific probes, allowing for further refinement of physical maps. Finally, this work underscores the need for high-quality internationally accessible germplasm collections. Our work benefited from excellent collections of *Avena* species housed in Canadian and American germplasm banks. Despite this, we have identified one species of paramount interest, *A. insularis*, which today is represented by only three known accessions.

#### Author contribution statement

HY: conducted experiments, analysed the data and co-wrote the manuscript; WAB: contributed to data analysis and interpretation of results; CPW: contributed to design and execution of experiments and interpretation of results; YP, TL RGL, Y-BF, AD, CJH, ENJ: contributed to experimental design, interpretation of results, and revisions of manuscript; BB: constructed GBS libraries and provided key advice on GBS analysis; YW, YP, and NAT: supervised HY; NAT conceived the study, analysed the data, and co-wrote the manuscript. All authors read and approved the final manuscript.

## Electronic supplementary material

Below is the link to the electronic supplementary material.

Online Resource 1 Details of germplasm assayed by GBS analysis (sheet 1) and list of accessions omitted because of potential misclassification (sheet 2) (XLSX 24 kb)

Online Resource 2 Raw data and GBS tags used in this work (shown in multiple worksheets, with explanations and table of contents provided on the first worksheet) (XLSX 37885 kb)

Supplementary material 3 (JPEG 709 kb)

Supplementary material 4 (JPEG 1794 kb)

Supplementary material 5 (JPEG 1050 kb)

Online Resource 6 Linear maps showing details of hexaploid haplotypes that match each species or ancestral group, and scaled version showing smoothed estimates of genome similarity based on a 30 cM sliding window. The scaled version was used in the construction of circle diagrams (Fig. 4, Online Resources 7 and 8) (XLSX 4261 kb)

Supplementary material 7 (JPEG 1517 kb)

Supplementary material 8 (JPEG 368 kb)
