# Supplementary figures and images for: High-density marker profiling confirms ancestral genomes of Avena species and identifies D-genome chromosomes of hexaploid oat
Source: Theor Appl Genet. 2016 Aug 13;129(11):2133–49. doi: 10.1007/s00122-016-2762-7 (PMC5069325; doi:10.1007/s00122-016-2762-7)

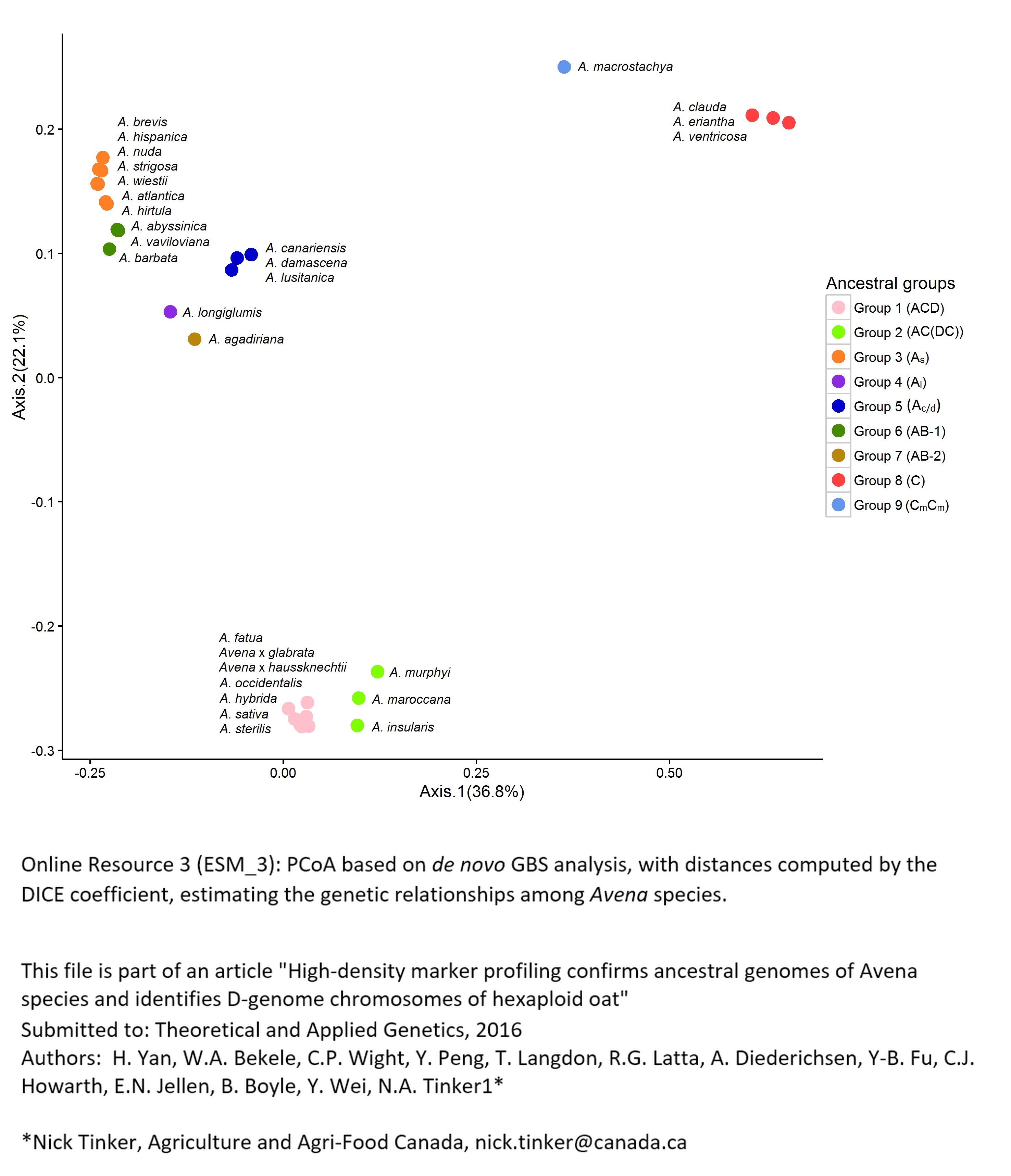

Supplement: Supplementary file 3 — Supplementary material 3 (JPEG 709 kb) [file 122_2016_2762_MOESM3_ESM.jpg]

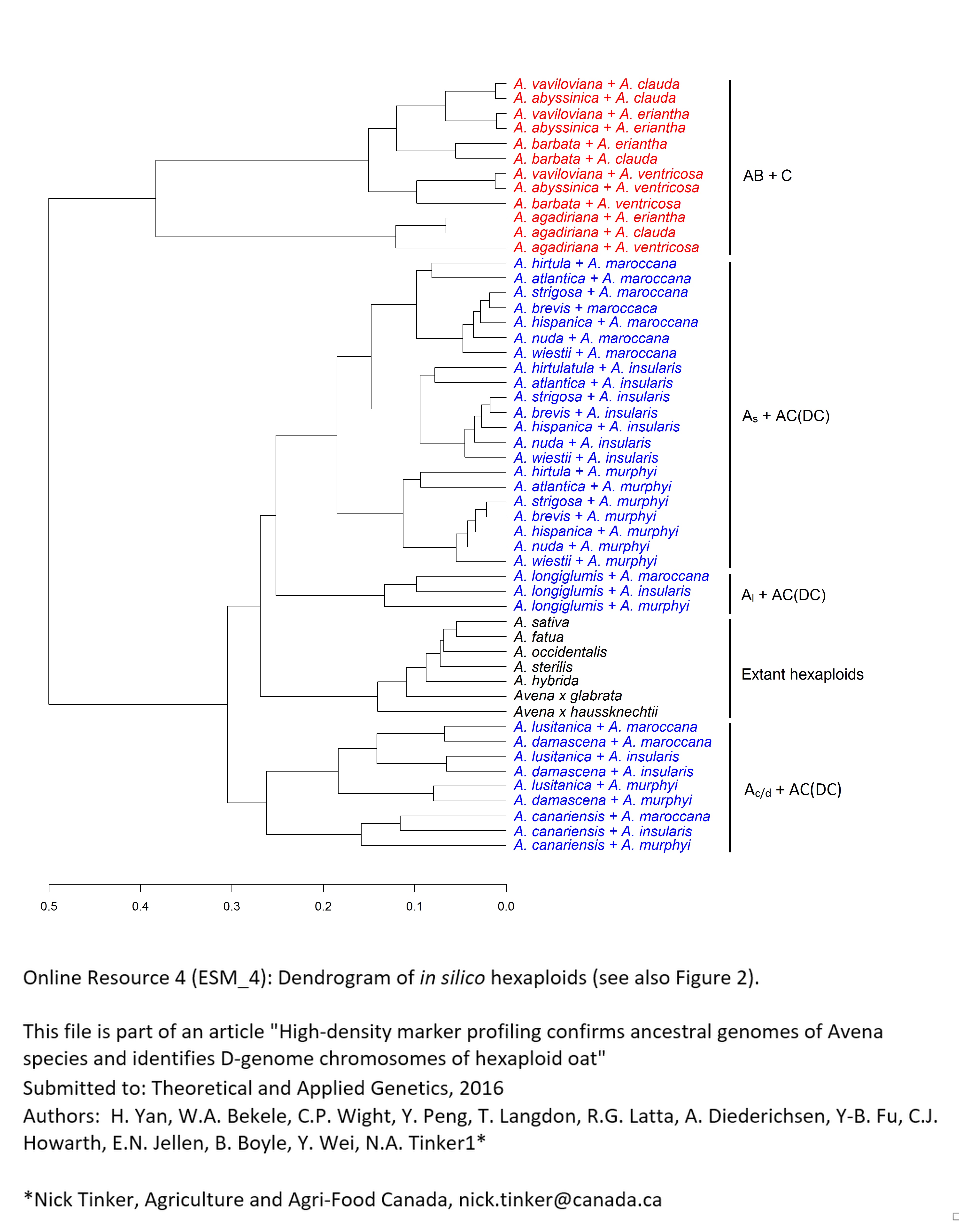

Supplement: Supplementary file 4 — Supplementary material 4 (JPEG 1794 kb) [file 122_2016_2762_MOESM4_ESM.jpg]

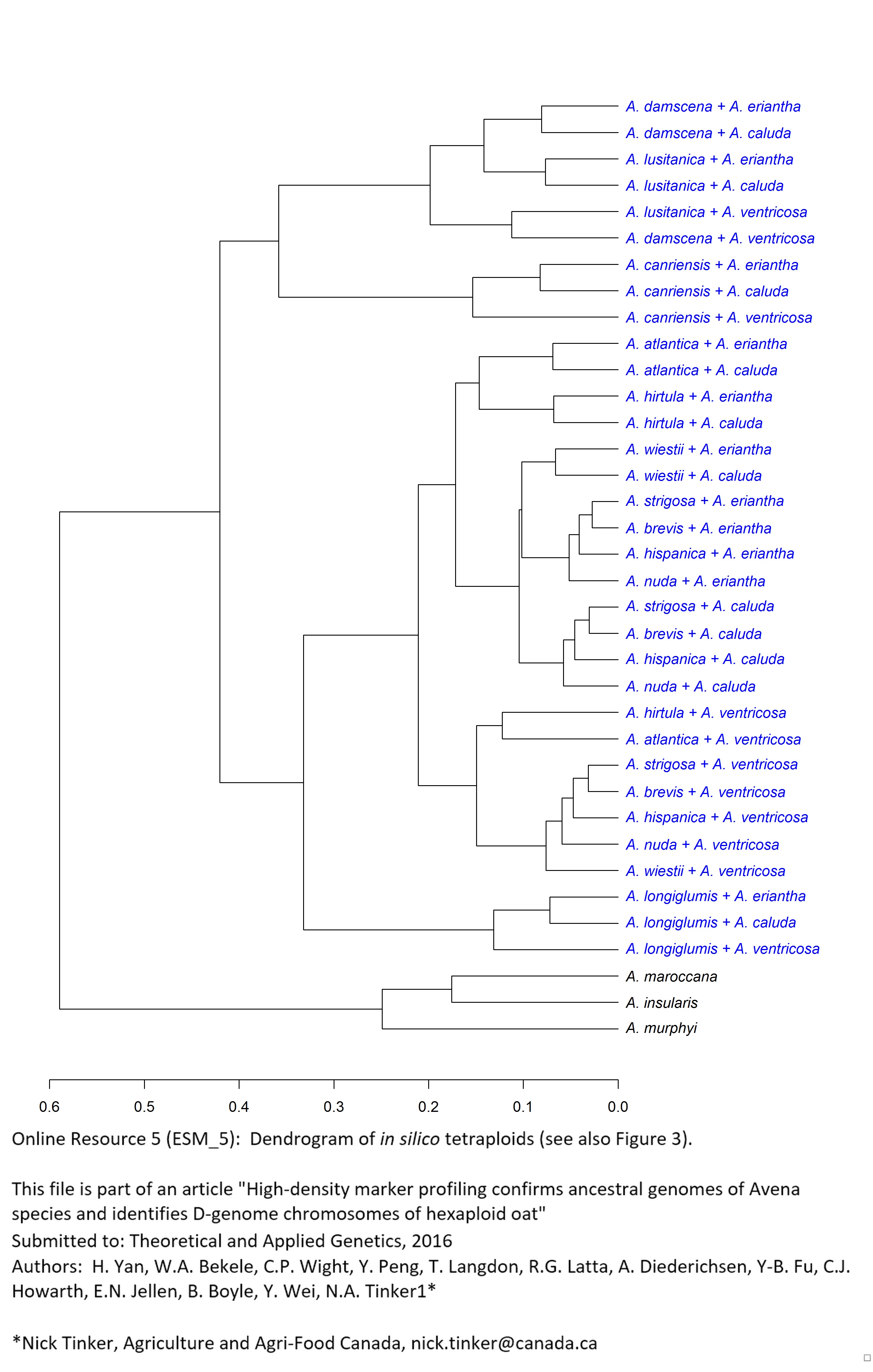

Supplement: Supplementary file 5 — Supplementary material 5 (JPEG 1050 kb) [file 122_2016_2762_MOESM5_ESM.jpg]

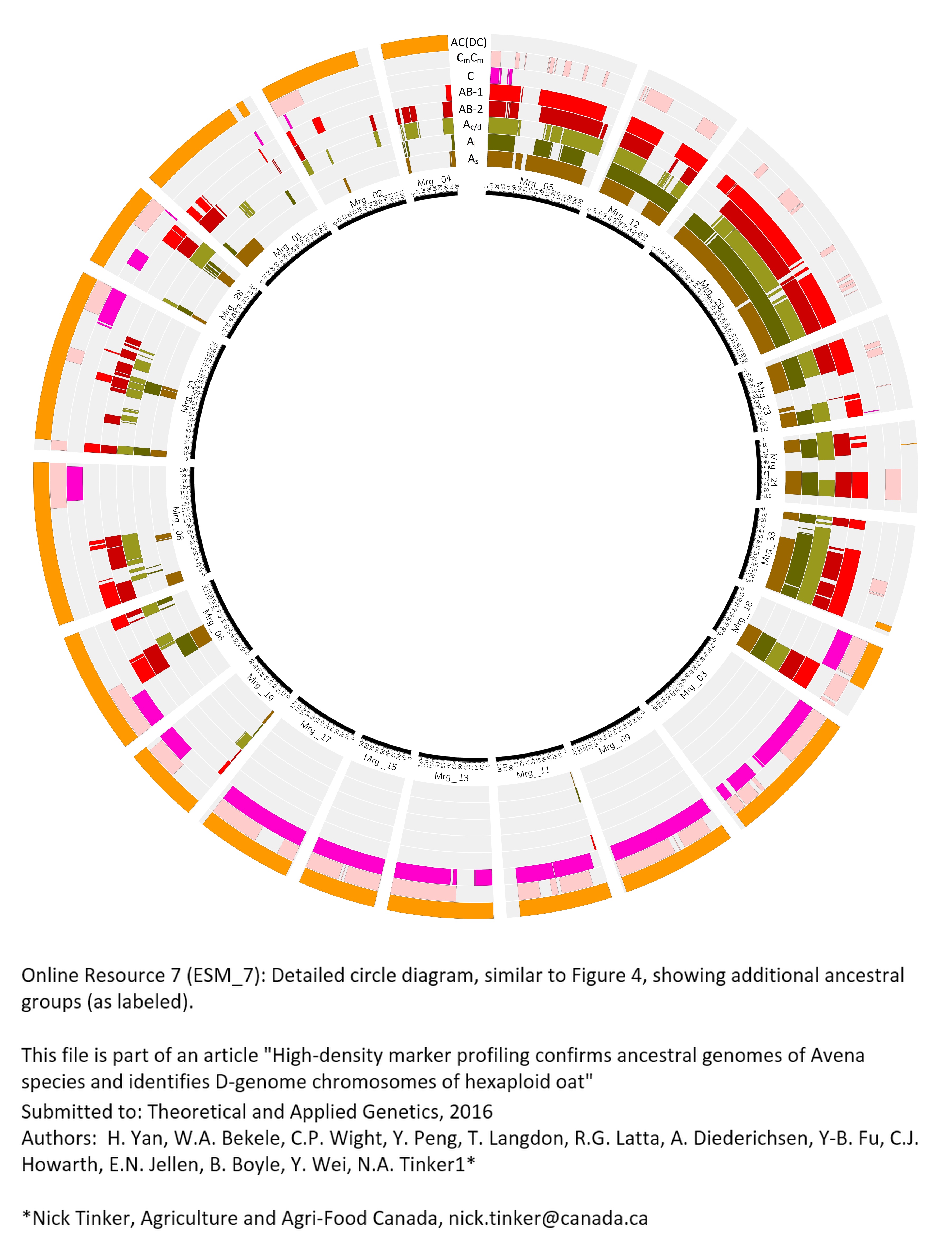

Supplement: Supplementary file 7 — Supplementary material 7 (JPEG 1517 kb) [file 122_2016_2762_MOESM7_ESM.jpg]

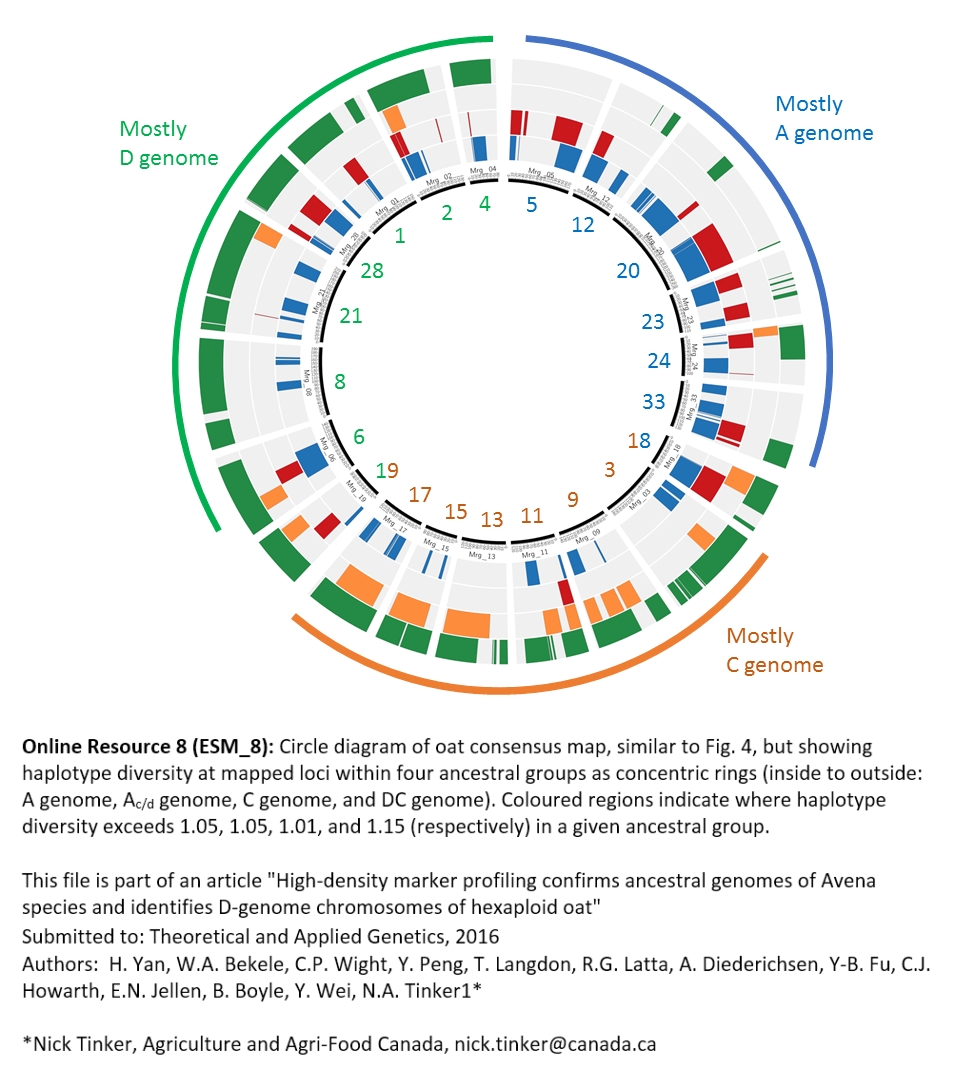

Supplement: Supplementary file 8 — Supplementary material 8 (JPEG 368 kb) [file 122_2016_2762_MOESM8_ESM.jpg]
